# Clinical and patient-reported outcome of implant restorations with internal conical connection in daily dental practices: prospective observational multicenter trial with up to 7-year follow-up

**DOI:** 10.1186/s40729-020-00211-z

**Published:** 2020-04-08

**Authors:** Karl-Ludwig Ackermann, Thomas Barth, Claudio Cacaci, Steffen Kistler, Markus Schlee, Michael Stiller

**Affiliations:** 1 Filderstadt, Germany; 2 Leipzig, Germany; 3 Munich, Germany; 4 Landsberg a. L., Germany; 5 Forchheim, Germany; 6 Department of Maxillofacial Surgery, Goethe University Frankfurt, Frankfurt, Germany; 7 Berlin, Germany

**Keywords:** Dental implant, Survival, Bone level change, Patient satisfaction, Observational multicenter study, Platform switching, Conical connection, Daily dental practice

## Abstract

**Background:**

The interpretation of the results of randomized clinical trials is often questioned in relation with daily circumstances in practices. This prospective observational multicenter study was instigated to reflect the need for information in real-life situations with dental implants with internal conical implant-abutment connection (Conelog implant system). The implants were followed up at least 5-year post-loading; survival analysis (Kaplan-Meier), changes of soft tissue, and bone level over time, as well as patient satisfaction were evaluated.

**Results:**

In total, 130 dental implants were placed in 94 patients (64 female, 30 male). Mean age of patients was 50.4 ± 13.7. At 5-year post-loading, 104 implants in 76 patients were available for evaluation. The cumulative implant survival rate was 96.6%. After an initial bone remodeling process post-surgery (bone loss of − 0.52 ± 0.55 mm), the bone level change remained clinically stable from loading to 5-year post-loading (− 0.09 ± 0.43 mm). Patient satisfaction surveyed by questionnaire (comfort, ability to chew and taste, esthetics, general satisfaction) steadily increased towards the end. At the last study follow-up, all the patients rated their general satisfaction as either very satisfied (87.5%) or satisfied (12.5%).

**Conclusion:**

The study implants have shown to be highly effective with reliable peri-implant tissue stability over the 5 to 7 years of observation for both single tooth restorations and fixed partial dentures while used in standard conditions in daily dental practice. The results obtained are comparable with those obtained in controlled clinical trials.

## Introduction

Many randomized controlled clinical trials have been published about dental implants. They have demonstrated long-term success in the rehabilitation of edentulous patients [1, 2] as well as patients with single or multiple teeth replacements [3, 4].

While this type of trials have an indispensable place in establishing a new product or a new operation technique regarding safety and efficacy, the results are viewed with a degree of caution by doctors in their daily practice because of the inherent weaknesses of controlled studies, such as very strict inclusion criteria, extremely motivated study patients, and extended treatment time. Hence, there is a growing interest to investigate the survival rates and adverse events (AE) encountered in daily practice. Systematic longitudinal studies reflecting the regular use of implants treatment were published to supplement the systematic assessment of implants [5]. Besides many retrospective studies [5–7], few observational clinical trials with a large number of patients are available [8–11].

While observational clinical trials are of high interest for daily practice, one drawback is their high dropout rate over the observed period in comparison to randomized controlled clinical trials, which may bias the results: a drop-out rate of 35% to nearly 50% has been published [8, 12]. The participating patients seem less willing to accept follow-up visits [13, 14] than in controlled randomized trials. To avoid any drop-out bias, it is therefore very important for treating doctors and their dental hygiene specialists to motivate patients to attend follow-up appointments.

The investigation of patient-related outcome measures (PROMs) have become important due to the fact, that clinical success has to be in line with the satisfaction of the patients with the restoration. The degree of individual patient satisfaction is the result of psychological and physiological factors. But the choice of which PROMs to use should be restricted to those most appropriate for the study question and at a minimum, these data should be gathered at two time points: at baseline and at a designated point post-treatment. Ideally, multiple assessments are desirable to discriminate short- versus long-term treatment effects [15–18].

Features of the chosen implant system for a study may also influence the outcome of the treatment: The degree of the manufacturer’s tolerances of implants with a conical implant-abutment connection heightens the risk of a mispositioning of the abutment, which cannot be corrected by repeated torqueing. Platform switching (PS) implants tend to have a protective effect on hard implant tissue outcomes, while implants with sandblasted, acid-etched surfaces allow for a shortened unloaded healing period [19–23].

This observational study was designed to document the clinical outcome of newly marketed dental implants and the supra-construction in the daily practice. As a primary objective implants survival rates over 5-year post-loading for single or multiple tooth replacement in the maxilla or the mandible were evaluated. Furthermore, these data were compared to the results of already published clinical studies and retrospective analyses. Secondary objectives included the evaluation of patients’ satisfaction with the restorations, changes of bone level over time, and the peri-implant soft tissue parameters such as plaque index (PI) and sulcus bleeding index (SBI).

## Methods

### Study design and population

This is an observational multicenter clinical study, approved by the ethics committee of the Freiburg ethics commission international (feci 010/1833). The study was planned and conducted according to the German medical devices law, the Declaration of Helsinki, good clinical practice, and the reporting is aligned with the STROBE statement. A minimum of 90 to 100 patients were planned to be included. Recruitment was performed in six centers (private practices) in Germany during a recruitment period of 16 months applying the following inclusion and exclusion criteria: Adult male or female patients aged ≥ 18 with one or several teeth missing in maxilla or mandible with sufficient bone at the planned implant sites were enrolled. Subjects with any contraindications included in the instructions for use of the implant system, heavy smokers (> 10 cigarettes or equivalents per day), pregnant, or breastfeeding women were excluded. After socket preservation and major bone augmentations, a period of at least 6 months had to elapse before implant surgery. All patients signed a written informed consent form. The study population consisted of 94 patients with 130 implants.

### Material and implant treatment

Conical dental implants with internal conical implant-abutment connections (Conelog Screw-Line implants; Camlog Biotechnologies GmbH, Basel, Switzerland) with diameters of 3.8 mm, 4.3 mm, and 5.0 mm, and lengths of 11 mm and 13 mm, and their corresponding prosthetic components including the PS concept were placed. The implant placement was performed in line with the manufacturer’s instructions for use of the implant system, and the treatment was done according to the study centers’ standards and the patients’ indications and has been described in detail elsewhere [10].

After submerged or transmucosal healing (at least 6 weeks when placed in class I, II, or III bone or 12 weeks in class IV bone), the implants were either restored with a provisional or directly with a definitive prosthesis based on the clinicians’ judgment. Implants were loaded with single crowns or fixed partial denture retained by a maximum of two implants.

**Fig. 1:**
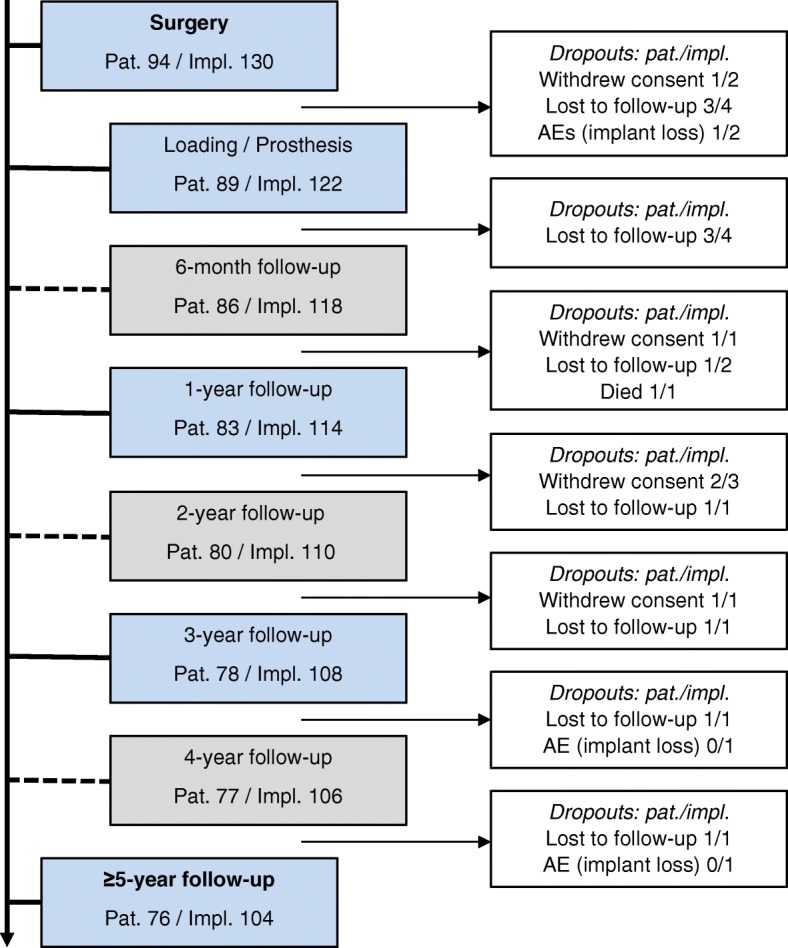
Study flow-chart: assessments and reason for dropouts. Study visits were done according to standard procedures in the respective study centers. Blue, mandatory visits; gray, optional visits

Patients were scheduled to follow-ups at 6 months, 1-, 2-, 3-, 4-, and 5-year post-loading for the assessment of the study parameter (Fig. 1). Depending on the investigators’ standard post-operative protocol, follow-up appointments were scheduled slightly differently: One center skipped the control visits at 6 months. Additionally, due to the observational character of the study and the patients’ willingness, a flexible scheduling was necessary. Regular oral maintenance care (dental hygiene session) was performed individually for every patient during the entire study period. X-rays were done as usual in the individual centers and photographs were taken. The oral health status was measured by assessing the plaque index and sulcus bleeding index, if routinely performed in the practice. All but one center documented the indices leading to representative results. And the patients filled a questionnaire asking for their satisfaction regarding comfort, appearance, ability to chew, ability to taste, and general satisfaction with their restoration at each visit (PROMs) [18, 24, 25].

### Study outcomes

The primary outcome was to assess implant survival of the implants 5-year post-loading. Secondary outcomes were changes of the bone level over time, evaluation of peri-implant soft tissue, as well as the evaluation of patients’ related outcome measures.

### Assessments, measurements

Prior to study start, all examiners met for calibration of the parameters. Implant survival and complications were documented at each study visit. Changes in crestal bone levels (BLC) were assessed measuring the distance implant shoulder to first visible bone contact (DIB) at the mesial and distal site of an implant on available radiographs, either peri-apical radiographs or orthopantomograms (OPTGs). The radiographs were not standardized throughout the study centers. Non-digital radiographs were digitized by scanning (Epson Perfection V700 Photo). The radiographs were calibrated and analyzed in an image-processing software (ImageJ 1.50i; http://imagej.nih.gov/ij). BLC were calculated as difference between surgery and loading, as well as between loading and 1-, 3-, and 5-year post-loading. Due to the observational study character, radiographs could not be collected systematically and therefore were not available for all patients and time points. The evaluation of soft tissue parameters and patient-related outcome measures regarding functional and esthetic outcome of the dental restorations are described in Figs. 4 and 5 respectively. Adverse events were documented throughout the study.

### Statistical analysis

The study data, soft and hard tissue parameters as well as the PROMs were descriptively analyzed using IBM SPSS V25.0 (IBM Corp., Armonk, NY, USA): Categorical variables were shown with frequencies and mean values were used for continuous variables. Survival analysis was performed using Kaplan–Meier method. Time of loading was the study baseline as per protocol, and the statistical unit was the dental implant.

## Results

### Demography

The study was started with 94 patients with 130 implants. At the end of the study (5-year post-loading), 76 patients with 104 implants were considered for analysis. Dropouts were distributed over the time of the study as described in Table 1. The majority of dropouts occurred early in the study phase. The reasons for the dropouts were variable as described in Fig. 1.

**Table 1:** Dropouts over observation time

Time period	Patients	Implants
Before loading	5	8
Loading-1 year	6	8
1-3 years	6	7
3-5 years	1	3
Total	18	26

Twelve percent of the patients (11 pat.) dropped out until 1-year post-loading. Six percent of the patients (6 pat.) dropped out between 1- and 3-year post-loading. One percent of the patients (1 pat.) dropped out between 3- and 5-year post-loading

The demographic and clinical parameters have been described in detail in [10]. Table 2 shows a further characterisation of patients. Four implants were placed immediately after tooth extraction while the majority of the implants were placed on healed extraction sites. On average, the implants were placed slightly subcrestally (0.32 ± 0.53 mm below crestal bone level). Two-stage surgery was applied in 66.7% of the cases, one-stage surgery in 33.3%. Twelve implants were loaded with a provisional beforehand. Single crowns were fixed on 103 implants, while a fixed partial denture was used in 10 cases (18 implants). The restorations were either cement-retained (81.4%) or screw-retained (18.6%).

**Table 2:** Demography of study population

Characteristics	Category	Total
Total patients/implants	*n*	94/130
	Center 1	9/17
	Center 2	18/26
	Center 3	20/26
	Center 4	14/16
	Center 5	18/26
	Center 6	15/19
Gender, *n* (%)	Male	30 (31.9)
	Female	64 (68.1)
Age at surgery, years	Mean ± SD	50.4 ± 13.7
	Range (min/max)	19.1-75.6
Age range distribution, *n* (%)	< 30 years	8 (8.5)
	30-45 years	22 (23.4)
	45-60 years	38 (40.4)
	60-75 years	25 (26.6)
	> 75 years	1 (1.1)
Tobacco use, *n* (%)	Non-smoker*	80 (85.1)
	Mild smoker (≤ 10/d)	14 (14.9)
General health status, *n* (%)	ASA P1	86 (91.5)
	ASA P2	8 (8.5)
Number of implants placed per patient, *n* (%)	1 implant	62 (66.0)
	2 implants	28 (29.8)
	3 implants	4 (4.3)
Distribution of implants in jaws, *n* (%)	Maxilla	59 (45.4)
	Mandible	71 (54.6)

*Former smoker counted as non-smoker (*n* = 16 (17%))

### Implant survival and complications

**Fig. 2:**
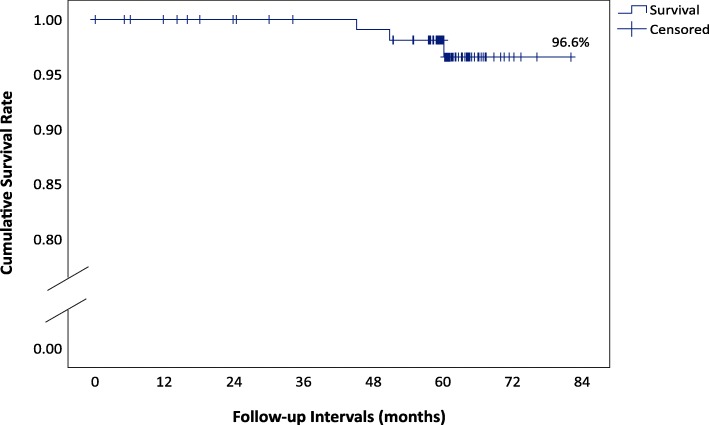
Kaplan-Meier cumulative survival rate

Two early implant losses in the healing phase were recorded, one due to infection and another due to radiolucency. Both implants had to be explanted prior to loading. After baseline, three implant losses were reported: two implants had to be extracted due to implant mobility (54 and 60 months post-loading), another due to peri-implantitis (45 months post-loading). The mean follow-up time was 62.3 months, the maximum 82 months. The cumulative proportion surviving rate up to 7-year post-loading was 96.6% (Kaplan-Meier, Fig. 2) with confidence interval lower bound 89.3% and upper bound 98.9%.

Further reported complications were peri-implant bone loss (> 2 mm) in three patients. Two of them started in the healing phase, the third in the follow-up period due to cement remains. All three could be treated or were still under treatment at study end. On the prosthetic level, three complications were reported as follows: two crown loosening and one chipping of crown. All crowns could be replaced with new crowns without further problems.

### Bone level changes

**Fig. 3:**
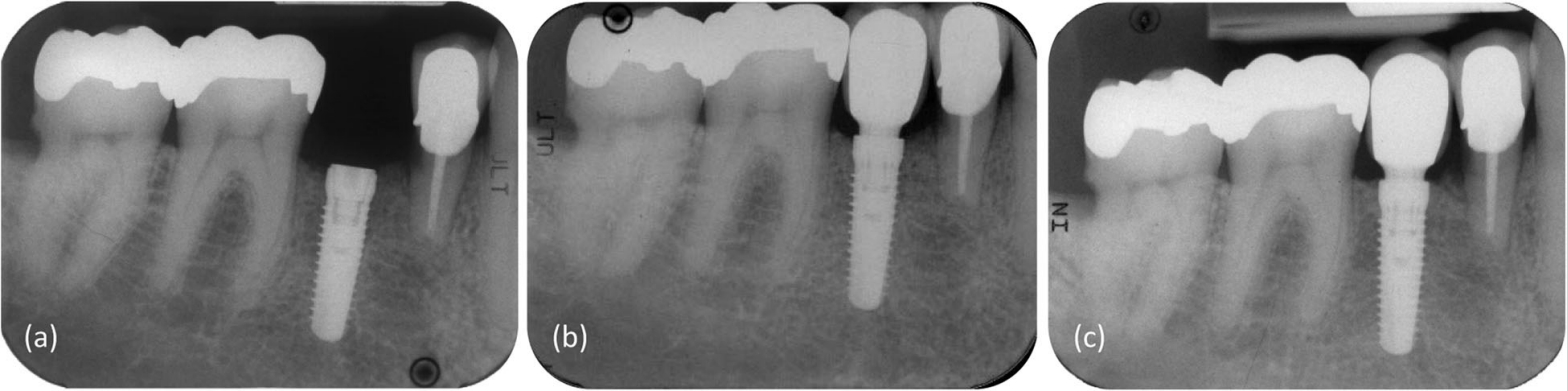
Standardized peri-apical radiographs representing the bone level changes at the implant level: immediately post insertion (**a**), at loading (abutment/crown placement) (**b**), and at 5-year post-loading (**c**)

Table 3 shows the mean bone level changes of the implants with available radiographs from insertion to 5-year post-loading. Bone remodeling around the implant was noticeable from surgery to loading, presenting a mean value of − 0.52 ± 0.55 mm. From loading to the 5-year follow-up, the mean change in crestal bone remained clinically stable (−0.09 ± 0.43 mm) (Fig. 3).

**Table 3:** Mean crestal bone level changes in mm

Bone level change	*n*	Mean	SD
Insertion-loading	103	− 0.52	0.55
Loading-1-year follow-up	93	− 0.04	0.37
Loading-3-year follow-up	90	− 0.04	0.40
Loading-5-year follow-up	86	− 0.09	0.43

For some patients no radiographs were available at the follow-up visits for various reasons (e.g., patient refusal for X-rays or not available from referring dentists)

Negative value indicates bone loss

Split into three groups, at 5-year post-loading, 15.1% of the implants were noted with a noticeable bone gain, 61.6% of the implants revealed a change in bone level of no clinical relevance (± 0.25 mm) and 23.3% of the implants experienced bone loss (> 0.25 mm).

### Soft tissue parameter

**Fig. 4:**
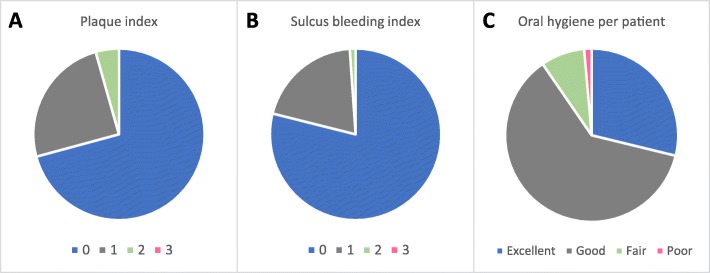
Soft tissue parameters at 5-year post-loading. **a** Plaque index: score 0, no plaque detected; score 1, plaque only recognized by running a probe across the smooth marginal surface of the implant; score 2, plaque seen by the naked eye; score 3, abundance of soft matter. **b** Sulcus bleeding index: score 0, no bleeding when a periodontal probe was passed along the gingival margin adjacent to the implant; score 1, isolated bleeding spot visible; score 2, blood formed a confluent red line on margin; score 3, heavy or profuse bleeding. **c** Oral hygiene assessed per patient

Oral hygiene status at 5-year post-loading is shown in Fig. 4. The general oral situation was subjectively assessed by the investigators at each visit after dental check-up based on the patients’ oral care behavior, tartar, and plaque. Before implantation, 31.9% of the patients presented an excellent, 66.0% a good, and 2.1% a fair oral hygiene. At the last follow-up, 28.8% of the patients presented an excellent, 61.6% a good, 8.2% a fair, and 1.4% a poor oral hygiene. This still excellent oral hygiene status at 5-year post-loading is congruent with the plaque and sulcus bleeding index: 96% of the implants were noted with no or only few plaque (scores 0 and 1), and 99% of the implants revealed no bleeding or only isolated bleeding spots upon probing (Fig. 4).

### Patient reported outcome measures

**Fig. 5:**
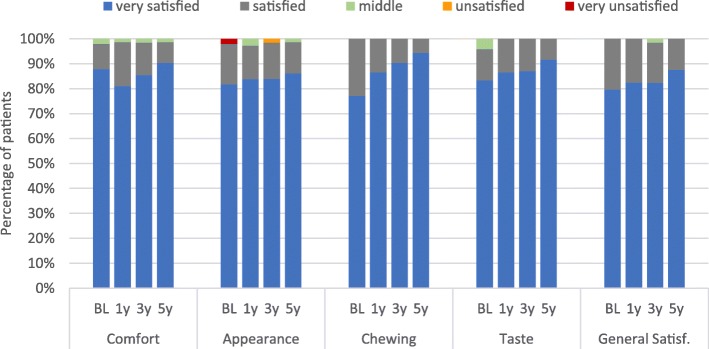
PROMs over 5 years: improvement of satisfaction from loading to study end

At the follow-up at 3-year post-loading, on a category scale of 1 (maximal satisfied) to 5 (very unsatisfied), 82.3% of the patients rated their general satisfaction as maximally satisfied, while 16.1% scored as satisfied. One patient (1.6%) rated his general satisfaction as neither satisfied nor unsatisfied due to esthetic problems induced by peri-implant hard and soft tissue recession. The same patient rated the appearance as unsatisfied (Fig. 5). At the last follow-up at 5 years, all the parameters of satisfaction improved to their maximum (general satisfaction: 87.5% of the patients were very satisfied and 12.5% satisfied).

## Discussion

Endosseous dental implants are a commonly accepted treatment procedure and showed high survival and success rates as well as good functional performance in numerous clinical trials and retrospective analyses, also for Conelog implants as for their specific implant surface [17, 23, 26]. However, in general, one could argue that results of controlled clinical studies do not reflect the real situation in daily dental practice, and every implant design and surface should be evaluated individually. Thus, this observational multicenter study was instigated to estimate the survival of these implants with internal conical implant-abutment connection in daily practice conditions with a great number of patients over 5 years. According to the study protocol, minimal exclusion criteria were applied in the selection of study participants alongside the usual contraindications and the technical procedure among the centers was not standardized to reflect daily practice. As a result, study participants recruited were heterogeneous as typically seen in daily dental practices to reflect the performance of these implants seen under these conditions.

After an observation period of 5- to 7-years post-loading, the implants demonstrated good performance with respect to implant survival. From the 122 implants restored, three implants (implant mobility and peri-implantitis) were lost, resulting in a cumulative proportion survival rate of 96.6% (Kaplan-Meier). Thus, although the selection of study participants was less stringent, the survival rate of implants and their corresponding prosthetic components in daily dental practices was very similar compared with randomized clinical studies over 5 years like Messias et al. [27], 96.6% with no difference between platform switching and platform matching abutments, or with the randomized controlled clinical study of Ioannidis et al. [4] resulting in a survival rate of 96.1% of the implants. In a meta-analysis over 5 years performed by Jung et al. [28] with more than 2000 patients, the survival of implants supporting single crowns was found to be 97.2%, and at 10 years 95.2%. The survival rate shown in this study is also comparable with other real-life data. An interesting approach in generally determining the efficacy of implants was undertaken by Seemann et al. [29]: In this retrospective study of the real-life return rate of 69,377 sold implants to the manufacturers all over Austria a return rate of 2.78%, i.e., survival rate of 97.22%, was demonstrated. But it has to be taken into account that this specific survival rate is based only on returned implants, which were considered by the treating doctors to be justifiable for reimbursement by the manufacturers.

Changes in crestal bone level are well-documented in the literature. Bone remodeling is reported to take place between surgery and loading. The reported changes are generally around 0.5 mm [17, 27, 30] but can reach more than 1 mm [31] in randomized or observational trials. The present study is well in accordance with these findings with its bone remodeling of − 0.52 ± 0.55 mm. Nearly 45% of the implants were placed subcrestally. These are associated with a remodeling of the crestal bone to the level of the implant shoulder which may be an explanation for the initial mean bone loss [10, 32, 33]. From loading to 5-year follow-up, clinically stable crestal bone levels at the implant shoulder were documented (− 0.09 ± 0.43 mm). Stable bone level or bone gain was noticed for 76.7% of the implants between loading and 5-year post-loading. A total of 23.3% of the evaluated implants were noted with a bone loss (12.8% with a loss > 0.5 mm). This bone gain corresponds well with the randomized clinical trial from Donati et al. [34], where 52% of the implants showed a bone gain over a 5-year observation period. These results are also in accordance with the controlled clinical studies published by Messias et al. [27] and Ioannidis et al. [4] and by the clinical study of Wennström et al. [35]. Additionally, the present results are consistent with the preliminary 1-year results with the same implant system published by Moergel et al. [17]. The integrated platform switching of the implants might additionally contribute to the stabilization of the bone as reported in several studies [21, 22, 27, 36].

Further contributing factors for the good survival data and bone level maintenance might be the regular follow-up controls with radiographs and patients’ care (plaque control, bleeding on probing, etc.). This allows an early detection of any focuses of inflammation and regular oral hygiene instructions. A recent meta-analysis by Lin et al. [37] showed a correlation between supportive care and peri-implant health. However, within an observational setting, study participants might be more difficult to follow up, especially when they are satisfied with their restorations and without any severe complications; they might tend to omit the control visits. This stresses the absolute importance of an excellent collaboration and guidance of the patients by their dental practices in terms of their continuing follow-up after the abutments are set. The very low drop-out rate of 19% in this purely observational multicenter study is in accordance with the results of randomized clinical studies published by others [18, 38] and is probably due to the stringent follow-up programs of the individual centers.

In recent years, patients’ needs have increased in terms of the esthetic and functional outcome of the dental restoration. PROMs have been reported in several studies and represent a well-described non-invasive method to measure patient’s satisfaction with these needs; however, they might present a lack of standardization [39, 40] due to the very subjective view on esthetics or functionality of the patients. Therefore, the chosen parameters considered to be important by the investigator might not correlate with the patients’ subjective satisfaction about the functional and esthetic results [41]. On the other hand, it is well-known from other medical fields that self-evaluation programs or forms for their health status increase the patient’s compliance with medication or treatment procedures considerably [42]. In analogy, one can assume that the filling out of the PROMs increases the motivation of the patients to adhere to oral hygiene with impact on the survival rate of the implants. In this observational study, patients’ satisfaction including esthetical and functional parameters as well as the oral hygiene status was evaluated. The data revealed that 87.5% of the patients were maximally satisfied and 12.5% satisfied at the 5-year follow-up appointment. The reason for these good results might be the excellent compliance of the patients in terms of oral hygiene which was also proven by the excellent results of the plaque and sulcus bleeding indices.

Observational studies often lack standardized treatment procedures. In this study different treatment protocols were applied beginning with the type of implantation (immediate versus delayed implantation), the healing procedure (submerged or transgingival healing), and the prosthetic restorations (screw- versus cement-retained; single crowns and fixed partial dentures). Additionally, the less stringent inclusion criteria than in (randomized) controlled clinical trials promote an increased heterogeneity of the study patients, but conversely may possibly reduce the risk of any bias toward more favorable outcomes. In summary, despite these possible limitations, the present results of implant survival, hard and soft tissue adaption as well as the patients’ satisfaction reflects the reality of implant treatment success in daily dental practice with reservation considering the defined indications.

## Conclusions

This prospective observational multicenter study demonstrated successful functional and esthetic outcomes of the study implant restorations (single tooth restoration, fixed partial dentures) with reliable peri-implant hard and soft tissue stability and high patients’ satisfaction. The results are comparable with the outcome of already published controlled randomized clinical studies and retrospective analyses confirming implants clinical appropriateness in daily dental practices. To assess the clinical performance of dental implants data of observational studies in daily dental practice complement the results achieved in controlled clinical studies.

## Data Availability

The datasets used and/or analyzed during the current study are available from the corresponding author on reasonable request.

## Abbreviations

AE: Adverse event
BLC: Bone level change
DIB: Distance implant shoulder to first visible bone contact
OPTG: Orthopantomogram
PI: Plaque index
PROMs: Patient reported outcome measures
PS: Platform switching
SBI: Sulcus bleeding index
